# Accessible and Adaptable Multiplexed Real-Time PCR Approaches to Identify SARS-CoV-2 Variants of Concern

**DOI:** 10.1128/spectrum.03222-22

**Published:** 2022-09-15

**Authors:** Ting Yan, Ye Xu, Rongrong Zheng, Xiaohong Zeng, Zehui Chen, Su Lin, Zihan Xia, Yiqun Liao, Yongyou Zhang, Qingge Li

**Affiliations:** a Engineering Research Centre of Molecular Diagnostics of the Ministry of Education, State Key Laboratory of Cellular Stress Biology, State Key Laboratory of Molecular Vaccinology and Molecular Diagnostics, School of Life Sciences, Faculty of Medicine and Life Sciences, Xiamen University, Xiamen, Fujian, China; b Xiamen Centre for Disease Control and Prevention, Xiamen, Fujian, China; University of Mississippi Medical Center

**Keywords:** SARS-CoV-2 VOCs, rapid genotyping, multiplex PCR, Omicron variant

## Abstract

Rapid identification and continuous surveillance of severe acute respiratory syndrome coronavirus 2 (SARS-CoV-2) variants are critical for guiding the response to the COVID-19 pandemic. Whole-genome sequencing (WGS) is a preferred tool for this aim, but many laboratories suffer from a lack of resources to support population-level sequencing. Here, we describe two PCR strategies targeting spike protein mutations to identify the Alpha, Delta, and Omicron variants. Signature mutations were selected using a dedicated bioinformatic program. The selected mutations in Alpha and Delta variants were detected using multicolor melting curve analysis (MMCA). Thirty-two mutations of the Omicron variant were targeted using the MeltArray approach in one reaction, which was able to detect the Omicron subvariants BA.1, BA.2, BA.3, and BA.4/5. The limits of detection varied from five to 50 copies of RNA templates/reactions. No cross-reactivity was observed with nine other respiratory viruses, including other coronaviruses. We validated the MMCA and MeltArray assays using 309 SARS-CoV-2 positive samples collected at different time points. These assays exhibited 98.3% to 100% sensitivity and 100% specificity compared with WGS. Multiplexed real-time PCR strategies represent an alternative tool capable of identifying current SARS-CoV-2 VOCs, adaptable for emerging variants and accessible for laboratories using existing equipment and personnel.

**IMPORTANCE** Rapid detection and mutation surveillance of SARS-CoV-2 VOCs is crucial for COVID-19 control, management, and prevention. We developed two rapid molecular assays based on the real-time PCR platform to identify important variants of concern, including the Omicron variant with a large number of mutations. Signature mutations were selected by an R program. Then, MMCA assays were established for Alpha and Delta variants, and a MeltArray assay targeting 32 mutations was developed for Omicron variant. These multiplexed PCR assays could be performed in a 96-well real-time PCR instrument within 2.5 h, offering a high-throughput choice for dynamic monitoring of SARS-CoV-2 VOCs in a standard microbiology laboratory.

## INTRODUCTION

Variants of the severe acute respiratory syndrome coronavirus 2 (SARS-CoV-2) have constantly emerged since the outbreak of the COVID-19 pandemic (1). Along with the sustained spread of the virus, certain variants have exhibited higher viral transmissibility and infectivity (2), stronger virulence (3), improved immune evasion, and resistance to neutralizing antibodies (4), resulting in clustered outbreaks and continuous waves of infection worldwide (5). Prompt identification and continuous surveillance of these variants are critical in guiding the outbreak response. Doing so would be guaranteed by the identification of variant-specific mutations and the development of appropriate detection strategies. Following the determination of the D614G variant in March 2020 (6), a series of variants of concern (VOCs) defined by the World Health Organization have emerged, including Alpha (B.1.1.7) (7), Beta (B.1.351) (8), Gamma (P.1) (9), Delta (B.1.617.2) (10), and the recent Omicron (B.1.1.529) lineage, which harbors unprecedentedly increased number of mutations and is now the predominant VOC (11).

Identification of SARS-CoV-2 variants primarily relies on whole-genome sequencing (WGS) (12), which can reveal all the mutations in the genome and identify the sequence characteristics of the virus, thereby allowing tracking of the viral transmission path (13). However, WGS is time-consuming, expertise-demanding, labor-intensive, and often associated with difficulties in monitoring large populations during periods of high incidence. Moreover, it is too expensive to implement in resource-limited settings (14). Thus, there is an urgent need for a complementary surveillance strategy that is more efficient and accessible to the vast majority of laboratories worldwide.

PCR has been widely used to detect SARS-CoV-2. Unlike WGS, nearly all laboratories offering this type of testing service have the necessary equipment and trained personnel to perform real-time PCR. Considering this need for accessible methods, we intended to provide identification schemes for VOCs based on real-time PCR. For this purpose, we developed a program that can generate variant-representative mutations by analyzing the SARS-CoV-2 sequences in the GISAID database. We then established assays to detect the chosen mutations that can represent the Alpha, Delta, and Omicron lineages, using either multicolor melting curve analysis (MMCA) (15) or MeltArray (16) approaches depending on lineage mutation count. These assays were used to dynamically monitor the prevalence of local VOCs.

## RESULTS

### Bioinformatics analysis.

We have continually monitored the mutation of SARS-CoV-2 since January 27, 2020. We retrieved the most current genome sequences of SARS-CoV-2 from GISAID for analysis using CovidShiny (https://github.com/zhanglabxmu/SARS-CoV-2). CovidShiny is based on an R open-source program, which contains functionalities to facilitate data visualization of global or regional mutation profiles in the genomic region targeted by sequence-based assays. This feature enables users to quickly evaluate and improve their molecular diagnostic assays. Using this tool, we were able to obtain the mutation frequencies from the viral genomes and select variant-specific mutations.

### Identification of Alpha and Delta variants via detection of the signature mutations by MMCA.

**FIG 1:**
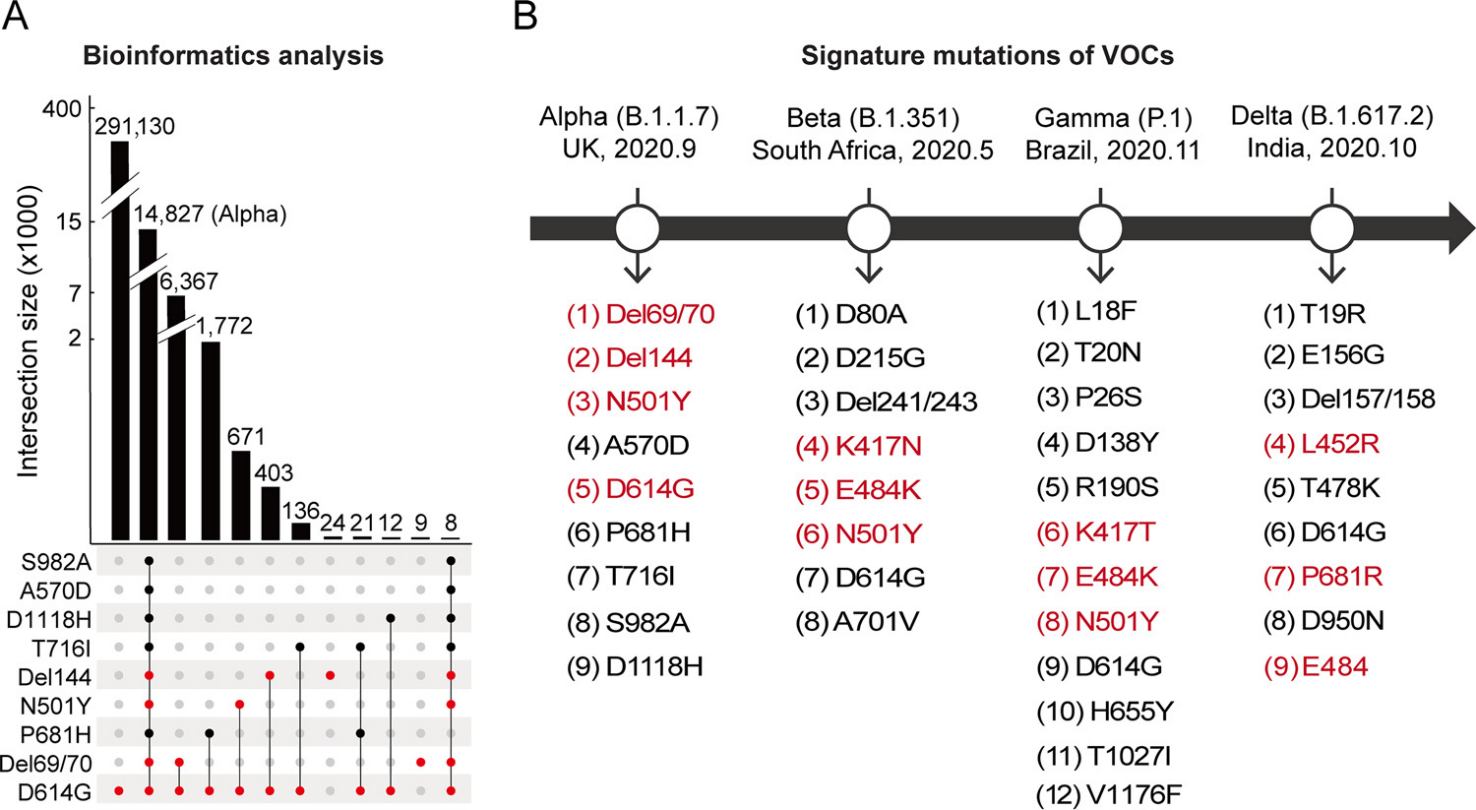
Results of signature mutations selection. (A) Bioinformatics analysis revealed that four mutations (Del69/70, Del144, N501Y, and D614G) could be used to identify the Alpha variant. (B) Feature mutations summary of four VOCs. The red letters indicate the chosen mutation sets for each VOC.

Using CovidShiny, four spike mutations, Del69/70, Del144, N501Y, and D614G, were chosen as the signature mutations for the Alpha variant (Fig. 1A). Similarly, signature mutations for Beta, Gamma, and Delta variants were generated (Fig. 1B).

**FIG 2:**
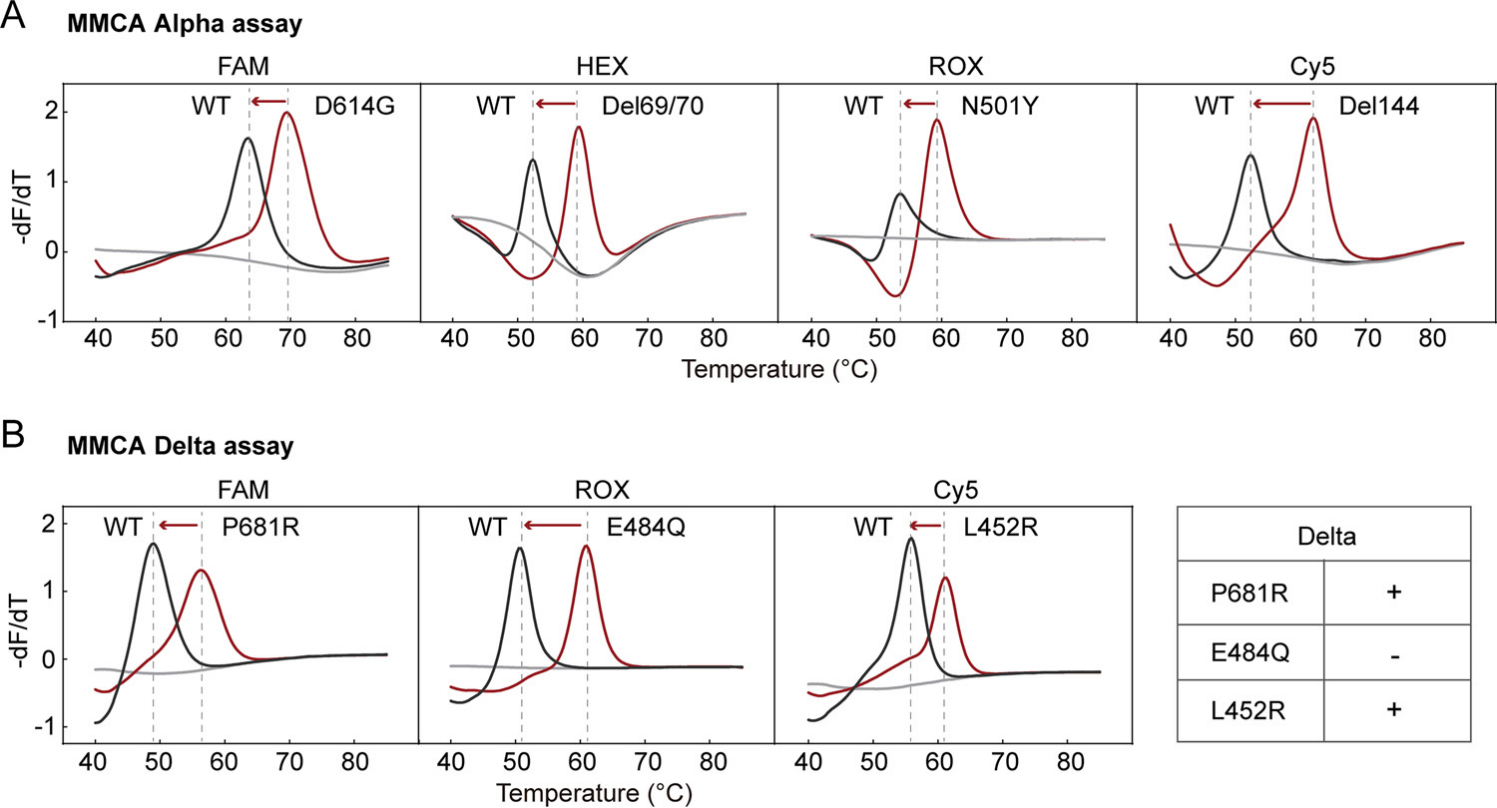
Identification of Alpha and Delta variants by detecting signature mutations using MMCA. (A) Typical results of the MMCA Alpha assay. (B) Typical results of the MMCA Delta assay. Four mutation-specific probes labeled with different fluorophores were used in the Alpha assay. Three mutation-specific probes labeled with different fluorophores were used in the Delta assay. Melting curves of mutant types and wild-type (WT) are shown according to the detection channels. Gray lines indicate no-template control (NTC). The inset panel shows the rule for identifying the Delta variant.

Multiplex PCR assay was designed to detect these signature mutations using multiple dually labeled, self-quenched probes in a reaction according to the MMCA scheme (15). To establish an MMCA Alpha assay, four probes were designed to be complementary to the mutant sequences; thus, the presence of the mutant would yield a higher *T_m_* value than that of the wild type. As such, the Alpha variant could be recognized by the presence of all four mutations (Fig. 2A). We evaluated the analytical performance of the MMCA assay using Armored RNA species containing the respective fragments of the wild-type and Alpha sequences. The limit of detection (LOD) was determined to be five copies per reaction for each of the four mutations (Table 1). The reproducibility experiment showed that the 3 SDs were less than 0.15°C (*n* = 10) for all *T_m_* values. A specificity study using nine other respiratory viruses showed no cross-reactivity. Using the MMCA Alpha assay, we screened 186 SARS-CoV-2 positive samples in a blinded manner. Among them, 67 samples collected before February 2020 had none of the four mutations (wild-type); 62 samples collected between March 2020 and April 2020 (D614G) and 57 samples collected between May 2021 and July 2021 (Delta variant) had the D614G mutation, but no Alpha variant was found. These results were in agreement with the WGS data.

**TABLE 1:** Analytical performance of VOCs assays

VOCs	Gene target	Channel	*T_m_* (°C)	Limit of detection
			(Mean ± 3SD)	(copies/reaction)
Alpha	Del69/70	HEX	59.35 ± 0.13	5
	Del144	Cy5	61.36 ± 0.12	5
	N501Y	ROX	60.24 ± 0.13	5
	D614G	FAM	70.04 ± 0.15	5
Delta	L452R	Cy5	61.43 ± 0.31	5
	E484Q	ROX	61.30 ± 0.18	5
	P681R	FAM	57.29 ± 0.26	5
Omicron	A67V	Atto 425	78.87 ± 0.19	50
	T95I	HEX	66.65 ± 0.13	50
	Del69/70	Cy5	68.28 ± 0.10	50
	G142D + Del143/145	Atto 425	74.75 ± 0.09	50
	N211I + Del212	ROX	60.61 ± 0.26	50
	Ins214EPE	Cy5	81.58 ± 0.18	50
	G339D	FAM	58.51 ± 0.28	50
	S371L	ROX	65.88 ± 0.13	50
	S373P	FAM	80.06 ± 0.25	50
	S375F	HEX	76.31 ± 0.13	50
	K417N	HEX	71.65 ± 0.09	50
	N440K	ROX	76.22 ± 0.10	50
	G446S	FAM	66.98 ± 0.19	50
	S477N	Cy5	86.21 ± 0.20	50
	T478K	Quasar 705	71.26 ± 0.21	50
	E484A	ROX	86.66 ± 0.13	50
	Q493R	FAM	62.82 ± 0.26	50
	G496S	Atto 425	70.89 ± 0.10	50
	Q498R	FAM	85.52 ± 0.20	50
	N501Y	Cy5	73.02 ± 0.10	50
	Y505H	FAM	71.66 ± 0.25	50
	T547K	Quasar 705	76.25 ± 0.21	50
	D614G	Atto 425	61.43 ± 0.18	50
	H655Y	HEX	86.23 ± 0.18	50
	N679K	ROX	81.23 ± 0.17	50
	P681H	Cy5	61.72 ± 0.26	50
	N764K	Quasar 705	80.68 ± 0.18	50
	D796Y	Quasar 705	86.30 ± 0.32	50
	N856K	ROX	53.72 ± 0.44	50
	Q954H	HEX	62.19 ± 0.16	50
	N969K	Atto 425	66.48 ± 0.09	50
	L981F	HEX	56.01 ± 0.15	50
	IPC	ROX	71.14 ± 0.08	50

To establish an MMCA Delta assay, three mutations (L452R, E484Q, and P681R) were identified by using three different fluorophore-labeled probes. The mutation E484Q was used to distinguish between the B.1.617.2 (Delta) and B.1.617.1 variants, with B.1.617.1 variant having the mutant version. Thus, the two mutations (L452R and P681R) and the E484 wild-type was used to identify Delta variant (Fig. 2B). Analytical evaluation studies showed that similar results were obtained as the above MMCA Alpha assay regarding the LOD, reproducibility (Table 1), and specificity. For clinical evaluation, we analyzed 186 SARS-CoV-2 positive samples in a blinded manner. The results showed that 67 samples collected before February 2020 (wild-type) and 62 samples collected between March 2020 and April 2020 (D614G) displayed no mutation signals, and 56 samples collected between May 2021 and July 2021 were Delta variants (L452R, P681R, and E484). Using WGS as the reference method, the MMCA Delta assay yielded 98.3% sensitivity and 100% specificity. One sample collected between May 2021 and July 2021 was identified as the Delta variant by WGS but not by the MMCA Delta assay. This sample had a lower *T_m_* value than that of the wild-type in the FAM channel (P681R), which was confirmed to have a single nucleotide polymorphism (SNP) at codon 680 (TCT>TTT) next to the P681R mutation, leading to decrease in the binding affinity of the probe and a lowered *T_m_* value.

### Identification of Omicron via full genotyping of 32 mutations by MeltArray.

**FIG 3:**
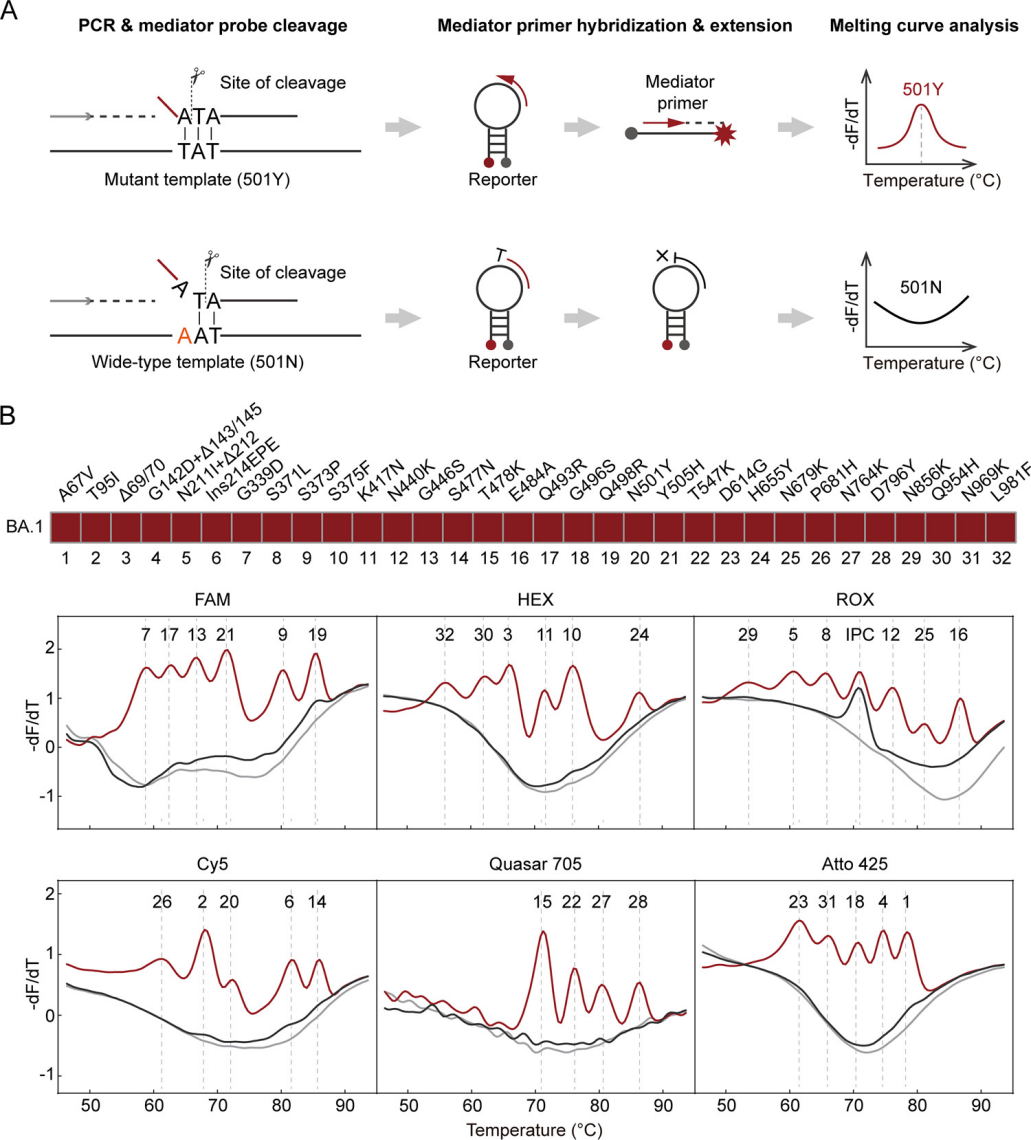
Identification of the Omicron BA.1 by the MeltArray assay. (A) The working principle of detecting single mutation by the MeltArray scheme. Taking N501Y mutation as an example, the mediator probe is perfectly matched the mutant template, and the mediator primer cleaved by the *Taq* DNA polymerase can extend along the molecular beacon reporter and emit a fluorescent signal, which can turn into a melting peak corresponding with the mutation. In contrast, when the probe binds to the wild-type template, due to the mismatch of bases, an extra base “T” is cut off, and the mediator primer cannot extend along the reporter and fail to generate a fluorescent signal. The presence or absence of a melting peak thus indicates whether the site is wild-type or mutant. (B) Results of the MeltArray assay for Omicron BA.1 identification. The upper panel lists the types of the 32 mutations, where “Δ” means deletion. The lower panel shows the detection results of the 32 mutations in Omicron BA.1 plus the IPC. Red lines indicate the melting curves from the BA.1 plasmid. Black lines indicate the melting curves of the wild-type plasmid. Gray lines indicate the melting curves of the NTC. The numbers above the melting peaks indicate the type of mutations.

The Omicron BA.1 variant has 32 mutations in the spike protein, far more than the number found in the Alpha and Delta variants. To examine all 32 mutations in one reaction, we used MeltArray, a multiplex mini-sequencing approach previously developed in our laboratory (16). In MeltArray, the *Taq* DNA polymerase cleaves the first base of the probe-binding region during the extension stage of PCR. We exploited this feature to identify the mutations at this locus. Using N501Y (AAT>TAT) as an example (Fig. 3A), a mediator probe was designed to match the mutant template. In the extension stage, the *Taq* DNA polymerase cleaves the mediator probe into a mediator primer with a terminal adenine that can bind to the molecular beacon reporter, which allows for the extension of the mediator primer to produce a fluorescent hybrid with defined melting temperatures unique to the target (501Y). If the mediator probe binds to the wild-type template, the enzyme will cleave one base after the mutated site and produce a mediator primer with terminal adenine and thymine, which cannot extend along the molecular beacon reporter and generate a fluorescent signal. Using multiple molecular beacon reporters labeled with different fluorophores, the overall number of targets was equal to the number of the reporters multiplied by the number of mediator primers per reporter. Our MeltArray reaction contained 32 mediator probes corresponding to 32 spike mutations and an additional probe targeting the conserved region of the N gene as an internal positive control (IPC). Given that the melting peaks are produced by the mutations and the IPC, a BA.1 template can generate 33 melting peaks with predefined *T_m_* values by MeltArray (Fig. 3B).

Next, we evaluated the analytical performance of the MeltArray assay using a BA.1 positive sample of known N gene copy numbers determined by ddPCR. All 33 targets were detected at concentrations ranging from 50 to 5,000 copies per reaction in 20 replicate reactions. The LOD was determined to be 50 copies per reaction (Fig. S1A). The reproducibility experiment showed that the 3 SDs for all the *T_m_* values ranged from 0.08°C to 0.44°C (*n* = 10) (Table 1), ensuring that all mutations were unambiguously distinguished. When tested with the wild-type template, no positive signals were observed, even at concentrations as high as 1 × 10^7^ copies/μL, demonstrating its high tolerance for the wild-type template. A specificity study using nine other respiratory viruses showed no cross-reactivity (Fig. S1B).

**FIG 4:**
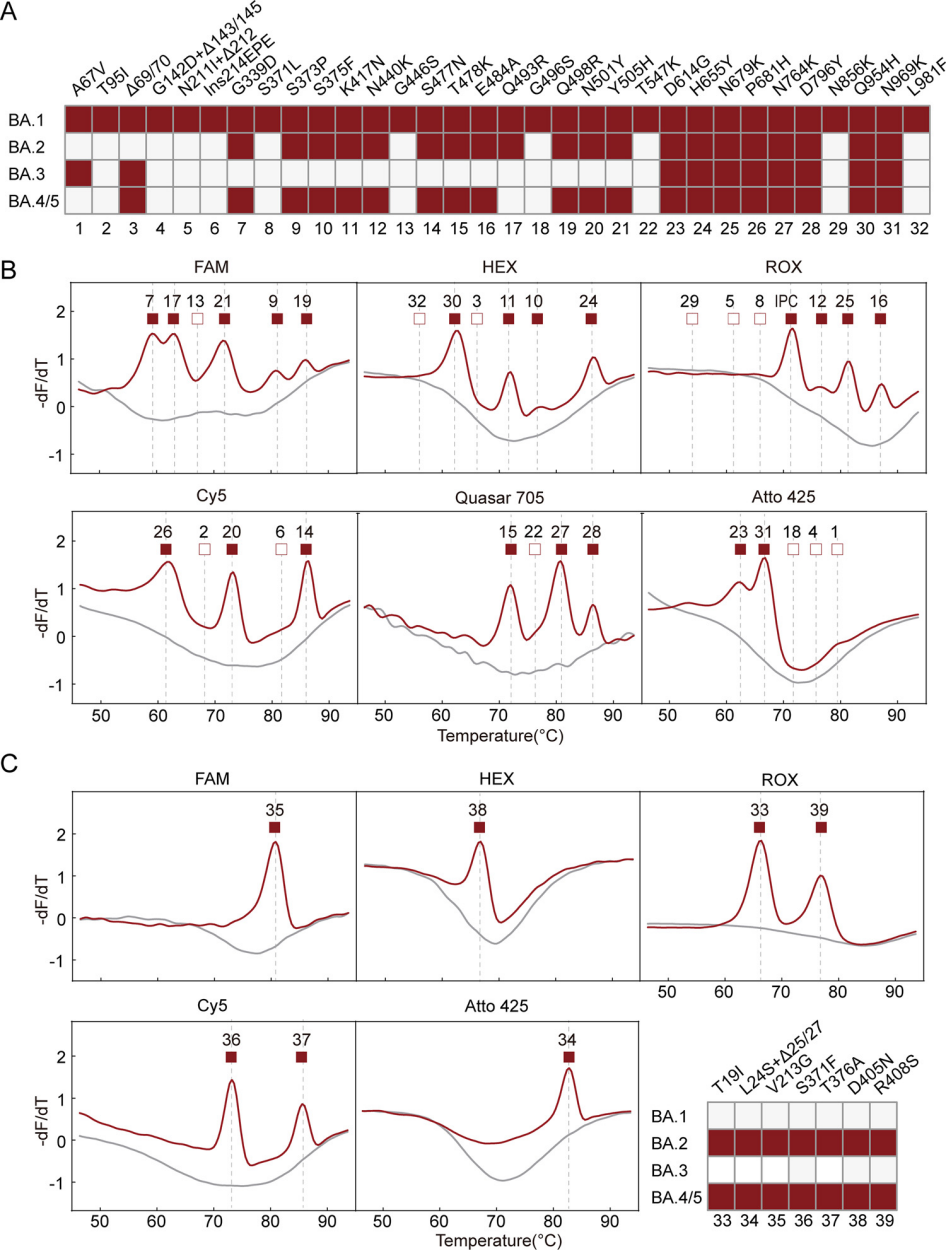
Identification of the Omicron subvariants using the MeltArray assay. (A) The mutation types present (solid square) and absent (empty square) in BA.1, BA.2, BA.3, and BA.4/5, where “Δ” means deletion. (B) Melting curve results of the BA.2 positive sample given by the 33-target MeltArray assay. Red lines indicate the results obtained from the BA.2 positive sample. Gray lines indicate the melting curves of the NTC. The numbers above the melting peaks indicate the type of mutations. (C) Melting curve results of the BA.2 positive sample given by the supplemental seven-target MeltArray assay. The inset panel shows the presence or absence of these seven mutations in different Omicron subvariants.

During this study, a new Omicron variant, BA.2, emerged and was followed by BA.3, BA.4, and BA.5 worldwide. We examined their spike mutation profile and found that they could be differentiated by the presence or absence of certain mutation types of the 32 mutations covered in our Omicron assay (Fig. 4A). To test this, a BA.2 positive sample was tested with the MeltArray assay, which displayed 21 melting peaks corresponding to the expected 20 BA.2 mutations and the IPC (Fig. 4B). This result demonstrated that the MeltArray assay designed for BA.1 could be used for BA.2, and may even be applicable to BA.3 and BA.4/BA.5 because of their distinct mutation patterns.

**FIG 5:**
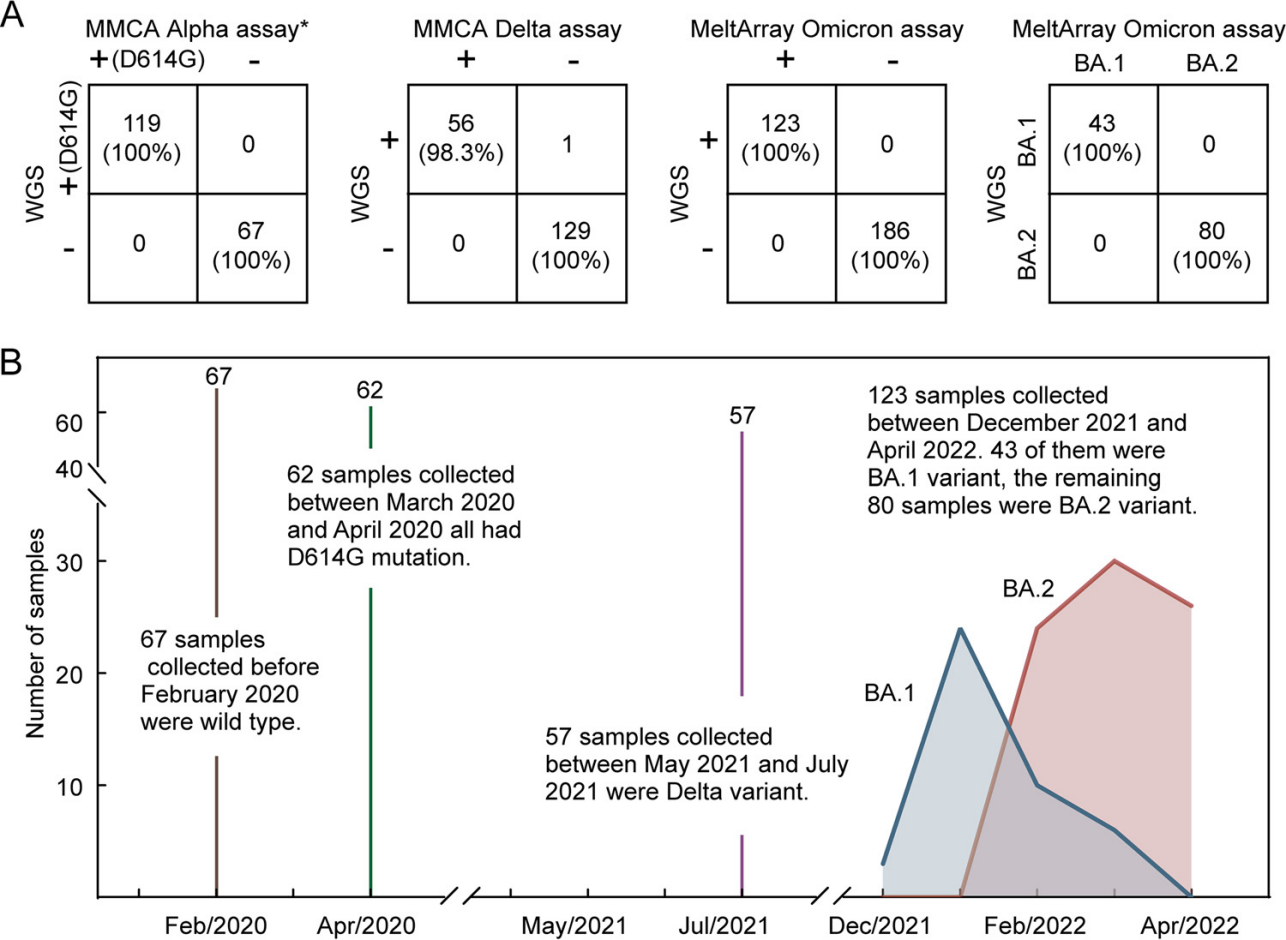
Summary of the detection results of VOCs in this study. (A) Agreements of the proposed assays with WGS in the detection of the Alpha, Delta, and Omicron variants. *In case of the Alpha assay, samples containing D614G mutation were used to calculate the clinical sensitivity because no other mutations were present. (B) A dynamic chart for the detection results of VOCs at different time points.

Using the MeltArray assay, we analyzed 309 SARS-CoV-2 positive samples in a blind manner. The results showed that 67 samples collected before February 2020 were wild-type with only IPC positivity, 62 samples collected between March 2020 and April 2020 displayed D614G and IPC positivity, 57 samples collected between May 2021 and July 2021 exhibited D614G, T478K, and IPC positivity, 123 positive samples collected between December 2021 and April 2022 were composed of 43 Omicron BA.1 (33 targets positive) and 80 Omicron BA.2 (21 targets positive). These results matched with those of the WGS (Fig. 5A). Our results showed that the BA.2 variant gradually replaced BA.1 variant over time, which was in accord with the local SARS-CoV-2 epidemiological data (Fig. 5B).

As both BA.2 and BA.4/BA.5 have seven extra mutations in the spike protein, an additional MeltArray assay targeting these seven mutations was established to complement the MeltArray Omicron assay. When testing the BA.2 sample, seven melting peaks were produced (Fig. 4C), further confirming the accuracy of the MeltArray Omicron assay.

## DISCUSSION

The continuous evolution of SARS-CoV-2 requires timely strategies for close surveillance (17). Here, we propose two strategies, MMCA and MeltArray assays, in response to different VOCs featuring varied mutation counts. These assays can both identify Alpha, Delta, and Omicron variants, and differentiate between all the major Omicron subvariants, including BA.1, BA.2, BA.3, and BA.4/BA.5. Notably, these two assays operate on a common platform, a real-time PCR thermal cycler, which is the most widely used instrument for detecting SARS-CoV-2 in clinical settings worldwide. With their single-tubed design, 96 samples can be processed in batch on a 96-well real-time PCR instrument within 2.5 h. These two assays offer an ideal choice for dynamic monitoring of different VOCs in a standard microbiology laboratory.

Various nonsequencing approaches have been described to screen for SARS-CoV-2 VOCs. The main strategy currently used is real-time PCR because of its ease of use, rapidness, cost-effectiveness, and, more importantly, easy access (18, 19). The correct selection of signature mutations representing a VOC is critical for successful identification. Several studies have employed mutations that are commonly found in different variants for assay development, but these mutations are not characteristic of a specific lineage and are often insufficient for variant discrimination (20–22). In this study, we developed an R program, CovidShiny, that automated the selection process for signature mutations by analyzing hundreds of thousands of sequences. The selected signature mutations proved to be reliable for identifying the early major VOCs, Alpha and Delta, in the beginning stages of the SARS-CoV-2 pandemic. We also identified signature mutations for other VOCs, such as the Beta and Gamma variants; however, these mutations were not assessed for their clinical performance due to their extreme rarity in our country.

The detection of one or a few target mutations using real-time PCR is relatively simple. To date, two strategies have been used for VOCs identification: allele-specific PCR (23) and melting curve analysis (21). The former uses a real-time detection mode and can be rapidly performed (<1.5 h); however, selection of allele-specific primers (23) or probes (24) is difficult and often challenging when multiple primers and probes work together in one reaction. The latter adopts an endpoint detection mode and has an extra melting analysis step (<30 min) after PCR. Establishing a melting curve analysis-based assay can be straightforward because of the simplicity of probe design, which benefits from the easy prediction of the probe *T_m_* shift (25). In this study, we chose an improved form of melting curve analysis, MMCA, as it permits the use of dually labeled, self-quenched probes that are easy to design, inexpensive to synthesize, and widely available (15). MMCA is particularly suitable for detecting multiple mutations if the total number of mutations does not exceed 10 in one reaction. Nevertheless, MMCA assays, like other melting curve analysis assays, can be difficult when unknown SNPs occur in the probe-binding region (26). Such SNPs may cause a *T_m_* shift, leading to misidentification or no identification. This was evidenced by the detection of 57 Delta variant samples, in which one sample carried an SNP at codon 680 next to the P681R mutation, leading to an abnormally lowered *T_m_* value and no sample identification. Despite its rarity, this incident reflected an inherent limitation of melting curve analysis-based assays, and any abnormal *T_m_* shift that occurs should be carefully examined.

When the number of target mutations exceeds 10, difficulties arise for the current real-time PCR strategies. For example, the Omicron variant contains 32 mutations in the spike protein, which is twice as many as the Delta variant (27). If detected by an allele-specific PCR or a MMCA assay, a large number of fluorogenic probes will be required to target each mutation. Multiple reactions will be necessary when run in a standard real-time PCR thermocycler equipped with four detection channels, resulting in lowered throughput and increased cost. Recently, Clark et al. described a protocol to detect SARS-CoV-2 VOCs by multiplex fragment analysis that could theoretically accommodate over 20 targets (28). However, an extra instrument for capillary electrophoresis and post-PCR operations are required. To overcome these difficulties, we chose the MeltArray scheme that could theoretically accommodate over 60 targets in one reaction using different combinations of fluorescent dyes and *T_m_* as labels (16). Due to the labeling nature of *T_m_*, an extra advantage of the MeltArray scheme is that it tolerates SNPs in the probe-binding region better than MMCA without causing a *T_m_* shift. Such a feature permits recognition of dozens of mutations in one reaction regardless of whether they are adjacent to each other. In this study, our MeltArray assay successfully detected 32 mutations of varied types in one reaction, allowing for the identification of all existing Omicron subvariants, including BA.1, BA.2, BA.3, and BA.4/5. Such performance differentiates the MeltArray assay from other approaches that only screen for one variant (29). Furthermore, the high multiplicity of the MeltArray scheme provides the MeltArray assay with sufficient capacity to accommodate more than 32 mutations. This aspect indicates that the assays can be updated to incorporate future SARS-CoV-2 VOCs. We noted that the LOD of the MeltArray assay was 50 copies per reaction, which was 10-fold higher than that of the MMCA assay. However, the MeltArray assay could correctly detect all SARS-CoV-2 positive samples, demonstrating that such analytical sensitivity is sufficient for routine use in identifying Omicron variants.

In summary, we developed a program for the batch analysis of the SARS-CoV-2 genome that can efficiently generate signature mutations representative of certain VOCs. We validated two types of real-time PCR-based genotyping assays for VOCs with different numbers of characteristic mutations. The MMCA assay can detect the Alpha and Delta variants, whereas the MeltArray assay can identify all the major Omicron subvariants. Both assays are characterized by easy access, ease of use, accuracy, and high throughput. Therefore, we anticipate that our adaptable and accessible strategies can find immediate use for routine monitoring of current SARS-CoV-2 VOCs and for the future development of new variant assays in microbiology laboratories worldwide.

## MATERIALS AND METHODS

### Plasmids, armored RNA species, and virus strains.

Plasmids containing either wild-type or SARS-CoV-2 variants spike sequences were synthesized by Amoy Nucleotide Biotechnology (Xiamen, China) and used to establish the detection assays. The corresponding armored RNA species containing the same sequences as the plasmids were prepared by Zeesan Biotech (Xiamen, China) and used for assay analytical performance evaluation. The concentrations of plasmids and Armored RNA species were determined by droplet digital PCR (ddPCR) using the TD1 Droplet Digital PCR System (Targeting One, Beijing, China).

Nine viral strains provided by Hexin Biotechnology (Guangzhou, China) were used to evaluate assay specificity: human coronavirus NL63, human coronavirus 229E, human coronavirus OC43, human rhinovirus 52, human parainfluenza virus 1, influenza virus A (H3N2), influenza virus B/Yamagata, influenza virus B/Victoria, and human adenovirus type 3.

### Sample collection, RNA extraction, and WGS.

A total of 309 SARS-CoV-2 RNA samples from nasopharyngeal swabs collected between February 2020 and April 2022 were used for clinical validation of the assay. RNA extraction was performed using the GeneRotex96 Nucleic Acid Extractor (Tianlong Science and Technology, Xi’an, China) according to the manufacturer's instructions. All samples were sequenced using the MGISEQ-200 platform (MGI Tech, Shenzhen, China) with the Pathogen Fast Identification system, according to the manufacturer’s instructions.

### MMCA assay procedures.

The reaction was performed in a 20-μL solution containing reverse transcription-PCR (RT-PCR) master buffer (Yeasen Biotechnology, Shanghai, China), 1 μL of enzyme mix (Yeasen Biotechnology), 5 μM forward primer, 50 μM reverse primer, 50 μM probe (Table S1), and 5 μL of RNA template. RT-PCR and melting curve analysis were performed using a SLAN 96S real-time PCR instrument (Hongshi Medical Technology, Shanghai, China) as follows: reverse transcription at 50°C for 15 min; denaturation at 95°C for 5 min; 50 cycles of 95°C for 15 s, 55°C for 15 s, 76°C for 20 s; 95°C for 1 min, 40°C for 3 min, followed by an increase from 40°C to 95°C (0.16°C/s). The fluorescence intensity was measured in four detection channels (FAM, HEX, ROX, and Cy5) at each step of the continuous temperature increase during melting curve analysis.

### MeltArray assay procedures.

Sample cDNA was obtained using the LF03 Reverse transcription kit (Zeesan Biotech) according to the manufacturer's instructions.

A 33-target MeltArray assay for Omicron variant identification was performed in a 25-μL solution containing PCR master buffer, 11 mM MgCl_2_, 0.5 mM dNTPs, 3 U *Taq* DNA polymerase (Zeesan Biotech), and 5 μL of cDNA template. Each reaction contained nine pairs of target-specific primers, 33 mediator probes (Table S2), and universal molecular beacon reporters (Zeesan Biotech). PCR and melting curve analysis were performed using the SLAN 96S real-time PCR instrument as follows: denaturation at 95°C for 2 min; 50 cycles of 95°C for 20 s, 60°C for 1 min; 35°C for 40 min, 95°C for 2 min, 40°C for 2 min, followed by an increase from 40°C to 95°C (0.16°C/s). The fluorescence intensity was measured in six detection channels (Atto 425, FAM, HEX, ROX, Cy5, and Quasar 705) at each step of the continuous temperature increase during the melting curve analysis.

A supplemental seven-target MeltArray assay for identifying signature mutations of the BA.2 variant was performed in a 25-μL solution containing PCR master buffer, 8 mM MgCl_2_, 0.4 mM dNTPs, 2 U *Taq* DNA polymerase (Zeesan Biotech), and 5 μL of cDNA template. Each reaction also contained three pairs of target-specific primers, seven mediator probes (Table S3), and universal molecular beacon reporters (Zeesan Biotech). PCR and melting curve analysis were identical to the Omicron assay process.

### Analytical evaluation.

Armored RNA species containing the SARS-CoV-2 spike gene fragment of the wild-type, Alpha, or Delta sequences were used for the analytical investigation of the MMCA assay. Clinical samples (BA.1 variant) of known concentrations predetermined by ddPCR and sequence information predetermined by WGS were used to evaluate the analytical performance of the MeltArray assay. To determine the LOD, a series of 10-fold diluted templates ranging from 1 to 10^3^ copies/μL was detected 20 times at each concentration. The lowest concentration with a positive result detection rate of 95% for all targets was considered the LOD. The reproducibility of the assay was determined by performing 10 replicate experiments in two batches (at a concentration of 10^2^ copies/μL), from which the 3-fold standard deviation (3 SD) for each averaged *T_m_* value was calculated. The specificity of the assay was evaluated by detecting nucleic acids (at a concentration of 10^7^ copies/mL) extracted from the nine other viruses listed above.

## Supplementary Material

Reviewer comments
