# Supplementary material for: Accessible and Adaptable Multiplexed Real-Time PCR Approaches to Identify SARS-CoV-2 Variants of Concern
Source: Microbiol Spectr. 2022 Sep 15;10(5):e03222-22. doi: 10.1128/spectrum.03222-22 (PMC9603638; doi:10.1128/spectrum.03222-22)
Supplement: Supplemental file 1 — Tables S1 to S3, Fig. S1. Download spectrum.03222-22-s0001.pdf, PDF file, 0.4 MB [file spectrum.03222-22-s0001.pdf]

Table S1. Primers and probes used in MMCA assays

Oligo name	Sequence (5′ → 3′) †	Concentration (μM)	Length (nt)
Del69/70-F	TCGCAAGCACTCACGTAGAGCTCAATTACCCCCTGCATACAC	5	42
Del69/70-R	GTCGCAAGCACTCACGTAGAGAGGACAGGGTTATCAAACCTC	50	42
Del69/70-P	HEX-CTCCATGCTATITCTGGGACCAATGGAG-BHQ1	50	37
Del144-F	TCGCAAGCACTCACGTAGAGCGAAGACCCAGTCCCTACTTATTG	5	44
Del144-R	GTCGCAAGCACTCACGTAGAGAGAAAAGGCTGAGAGACATATTCA	50	45
Del144-P	CY5-ACCATTTTTGGGTGTTTACCAC-BHQ1	50	31
L452R-F	TCGCAAGCACTCACGTAGAGTCTTGATTCTAAGGTTGGTGGT	50	42
L452R-R	GTCGCAAGCACTCACGTAGAGAGCTGGTGCATGTAGAAGTTC	50	42
L452R-P	CY5-CCTTACCGGTATAGATTGTTTAGGAAGG-BHQ1	50	37
E484Q-F	TCGCAAGCACTCACGTAGAGTCTTGATTCTAAGGTTGGTGGT	5	42
E484Q-R	GTCGCAAGCACTCACGTAGAGAGCTGGTGCATGTAGAAGTTC	50	42
E484Q-P	ROX-aCTTGTAATGGTGTTCAAGGTT-BHQ2	50	22
N501Y-F	TCGCAAGCACTCACGTAGAGCTGAAATCTATCAGGCCGGT	5	40
N501Y-R	GTCGCAAGCACTCACGTAGAGAGCTGGTGCATGTAGAAGTTC	50	42
N501Y-P	ROX-TCCCACTTATGGTGTTGGTT-BHQ1	50	29
D614G-F	TCGCAAGCACTCACGTAGAGCCGTGATCCACAGACACTTG	5	40
D614G-R	GTCGCAAGCACTCACGTAGAGACACGCCAAGTAGGAGTAAG	50	41
D614G-P	FAM-TATCAGGGTGTTAACTGCA-BHQ1	50	28
P681R-F	TCGCAAGCACTCACGTAGAGCAGGTATATGCGCTAGTTATC	50	41
P681R-R	GTCGCAAGCACTCACGTAGAGAGTGTAGGCAATGATGGATTGAC	50	44
P681R-P	FAM-CGACTCAACTAATTCTCGTCG-BHQ1	50	30

†Underlined nucleotides indicate locked nucleic acids

Table S2. Primers and probes used in the 33-target Omicron assay

Oligo name	Sequence (5′ → 3′) †	Concentration (nM)	Length (nt)
F1	GCAAGCCCTCACGTAGCGAAGTGTTTATTACCCTGACAAAGT	40	42
R1	GCAAGCCCTCACGTAGCGAATGAACTCACTTTCCATCCA	40	39
F2	GCAAGCCCTCACGTAGCGAATGGACCTTGAAGGAAAACA	20	39
R2	GCAAGCCCTCACGTAGCGAACACCAGGAGTCAAATAACTTC	20	41
F3	GCAAGCCCTCACGTAGCGAATACGTTGAAATCCTTCACTG	40	40
R3	GCAAGCCCTCACGTAGCGAACTGCATAGACATTAGTAAAGCA	40	42
F4	GCAAGCCCTCACGTAGCGAAGGTGATGAAGTCAGACAAATC	40	41
R4	GCAAGCCCTCACGTAGCGAACAAACAGTTGCTGGTGC	40	37
F5	GCAAGCCCTCACGTAGCGAAGTTTGTGGACCTAAAAAGTCT	20	41
R5	GCAAGCCCTCACGTAGCGAAATCTGCATGAATAGCAACAG	20	40
F6	GCAAGCCCTCACGTAGCGAATCTACAGGTTCTAATGTTTTTCA	20	43
R6	GCAAGCCCTCACGTAGCGAAGGTATGGCAATAGAGTTATTAGAG	20	44
F7	GCAAGCCCTCACGTAGCGAAGAATGCAGCAATCTTTTGTT	20	40
R7	GCAAGCCCTCACGTAGCGAATTGAGCAATCATTTCATCTGT	20	41
F8	GCAAGCCCTCACGTAGCGAAGCTATTGGCAAAATTCAAGAC	20	41
R8	GCAAGCCCTCACGTAGCGAACAAACTTTGAAGTCTGCCT	20	39
F9	GCAAGCCCTCACGTAGCGAACTGCTGCTTGACAGATTGA	40	39
R9	GCAAGCCCTCACGTAGCGAACCTCAGCAGCAGATTTCTT	40	39
Universal primer	GCAAGCCCTCACGTAGCGAA	1600	20
A67V-P	CAGTGCTGCTCACATGGAACCAAGTAACATTGGAAAAGAAAGGT-C_7NH_2	200	50
T95I-P	CTCTCTCTGGTCTTGAGAAGTCTAACATAATAAGAGGCTGGATTTTTGG-C_7NH_2	100	55
Del69/70-P	TTCCACATCGTATCTCTGGGACCAATGGTACTAAGAG-C_7NH_2	80	43
G142D-P	TGTCTCGGTACTCTGGTCCAAAAATGGATCATTACAAAATTGAAATTCA-C_7NH_2	60	55
N211I-P	ACATCTCACACTTCACTATAATAGGCGTGTGCTTAGAATATATTTTAAAATAACC-C_7NH_2	100	61
Ins214EPE-P	GCTCCTCCTGTGAGCCAGAAGATCTCCCTCAGGGT-C_7NH_2	60	41
G339D-P	TCACACTGATCTGTCAAAAGGGCACAAGTTTGTAATATTAGGAAATCTAA-C_7NH_2	100	56
S371L-P	CACCTCTCACAGATTATATAGGACAGAATAATCAGCAACACAGTTGCTGATT-C_7NH_2	60	58

Continued

S373P-P	CCGTGCTCACCCATTTTICACTTTTAAGTGTTATGGAGTGTCTCC-C_7NH_2	100	51
S375F-P	CCGTGTCCCTCACTTTTAAGTGTTATGGAGTGTCTCCTACTAAAT-C_7NH_2	200	51
K417N-P	CTCTGACTCTATTGCTGATTATAATTATAAATTACCAGATGATTTTACAGGC-C_7NH_2	500	58
N440K-P	AGCGCTCTCCGCTTGATTCTAAGGTTAGTGGTAATTATAATTACCTGT-C_7NH_2	40	54
G446S-P	CCTCACTCTGAGTGGTAATTATAATTACCTGTATAGATTGTTTAGGAAGT-C_7NH_2	80	56
S477N-P	CCTGGTGAGCTTACCGGCCTGATAGATTTCAGTTGAAATATCTCTCACCAGG-C_7NH_2	60	58
T478K-P	CGTGTGACTGAACCTTGTAATGGTGTTGAAGGTTTTAATTGTTACTT-C_7NH_2	16	53
E484A-P	GTCCACACTGTCAGGTTTTAATTGTTACTTTCCTTTACAATCATATGGTT-C_7NH_2	40	56
Q493R-P	CACTCTGATCTGATCATATGGTTTCCGACCCACTAATGGTG-C_7NH_2	440	47
G496S-P	GGTACTCTCTGAGTTTCCGACCCACTAATGGTGTTGGTTAC-C_7NH_2	200	47
Q498R-P	CCCTCCTGCCTGACCCACTIATGGTGTTGGTTACCAACCATACAGAGTA-C_7NH_2	140	55
N501Y-P	CTGACTTCCAAGTGGGTTGGAAACTATATGATTGTAAAGGAAAGTA-C_7NH_2	400	52
Y505H-P	TCACGTGTGGTCGTGCACCAACCATACAGAGTAGTAGTACTTTCTTTTGAAC-C_7NH_2	200	58
T547K-P	AGCCCCGTGTTTAAACCATTGAAGTTGAAATTGACACATTTGTTTTTAACCA-C_7NH_2	400	58
D614G-P	CTCAGACTGTCTATTCCCTGATAAAGAACAGCAACCTGGTTAGAAG-C_7NH_2	100	52
H655Y-P	CAGCCGTCCGATTCAGCCCCTATTAAACAGCCTGC-C_7NH_2	400	41
N679K-P	ACACTGTCCAGCTTAGTCTGAGTCTGATAACTAGCGCATATACCT-C_7NH_2	200	51
P681H-P	ATCGTTGAGTTTTGAGAITTAGTCTGAGTCTGATAACTAGCGCATATACCTCAACGAT-C_7NH_2	400	64
N764K-P	GACCGGTGCACACGTGCTTTAACTGGAATAGCTGTTGAACAA-C_7NH_2	100	48
D796Y-P	CCCACCTCCGATTTAATTGGTGGTGTTTTGTAAATTTGTTTGACTT-C_7NH_2	400	52
N856K-P	TCGCACTTCTTTTTTTAAACTTTTGTGCACAAATGAGGTCTCTAGCAG-C_7NH_2	200	54
Q954H-P	CTCTGGTCTATCTAATGCACAAGCTTTAAACACGCTTGTTAAAC-C_7NH_2	120	50
N969K-P	CTCTCTGACTGTCTTTGGAGCTAAGTTGTTTAACAAGCGTGTTTAAAGGTCAGAGAG-C_7NH_2	160	63
L981F-P	GTCTATCTTTTATTTTCACGTCTTGACAAAGTTGAGGCTGAAG-C_7NH_2	400	49
IPC-P	TCATCACACCTAAAGGCCAACAACAACAAGGCCAAAC-C_7NH_2	16	43

†Underlined nucleotides indicate locked nucleic acids

Table S3. Primers and probes used in the seven-target supplemental assay

Oligo name	Sequence (5′ → 3′) †	Concentration (nM)	Length (nt)
F1	GCAAGCCCTCACGTAGCGAATGTTTGTTTTTCTTGTTTTATTGC	40	44
R1	GCAAGCCCTCACGTAGCGAAATTAAATGGTAGGACAGGGT	40	40
F2	GCAAGCCCTCACGTAGCGAATGGACCTTGAAGGAAAACA	40	39
R2	GCAAGCCCTCACGTAGCGAACACCAGGAGTCAAATAACTTC	40	41
F3	GCAAGCCCTCACGTAGCGAATTAACGCCACCAGATTTG	40	38
R3	GCAAGCCCTCACGTAGCGAAGCCTGTAAAATCATCTGGTAA	40	41
Universal primer	GCAAGCCCTCACGTAGCGAA	800	20
T19I-P	CACCTCTCACATAAGATTAACACACTGACTAGAGACTAGTG-C_7NH_2	100	47
L24SΔ-P	GGGACCTGACATCATACACTAATTCTTTCACACGTGGTGTT-C_7NH_2	500	47
V213G-P	CCGTGCTCACCTGGCGTGATCTCCCTCAGGCACGG-C_7NH_2	160	41
S371F-P	CTGACTTCCACAAATTATATAGGACAGAATAATCAGCAACACAGTTGCT-C_7NH_2	400	55
T376A-P	GTGAGCTGCTCGAAAAATGGTGCGAAATTATATAGGACAGAATAATCAGCTCAC-C_7NH_2	400	60
D405N-P	CTCTCTCTGGTACCTCTAATTACAAATGAATCTGCATAGACATACCAGAGAGAG-C_7NH_2	500	60
R408S-P	AGCGCTCTCCGCTGACTTCATTACCTCTAATTACAAATGAATCTGCATA-C_7NH_2	200	55

†Underlined nucleotides indicate locked nucleic acids

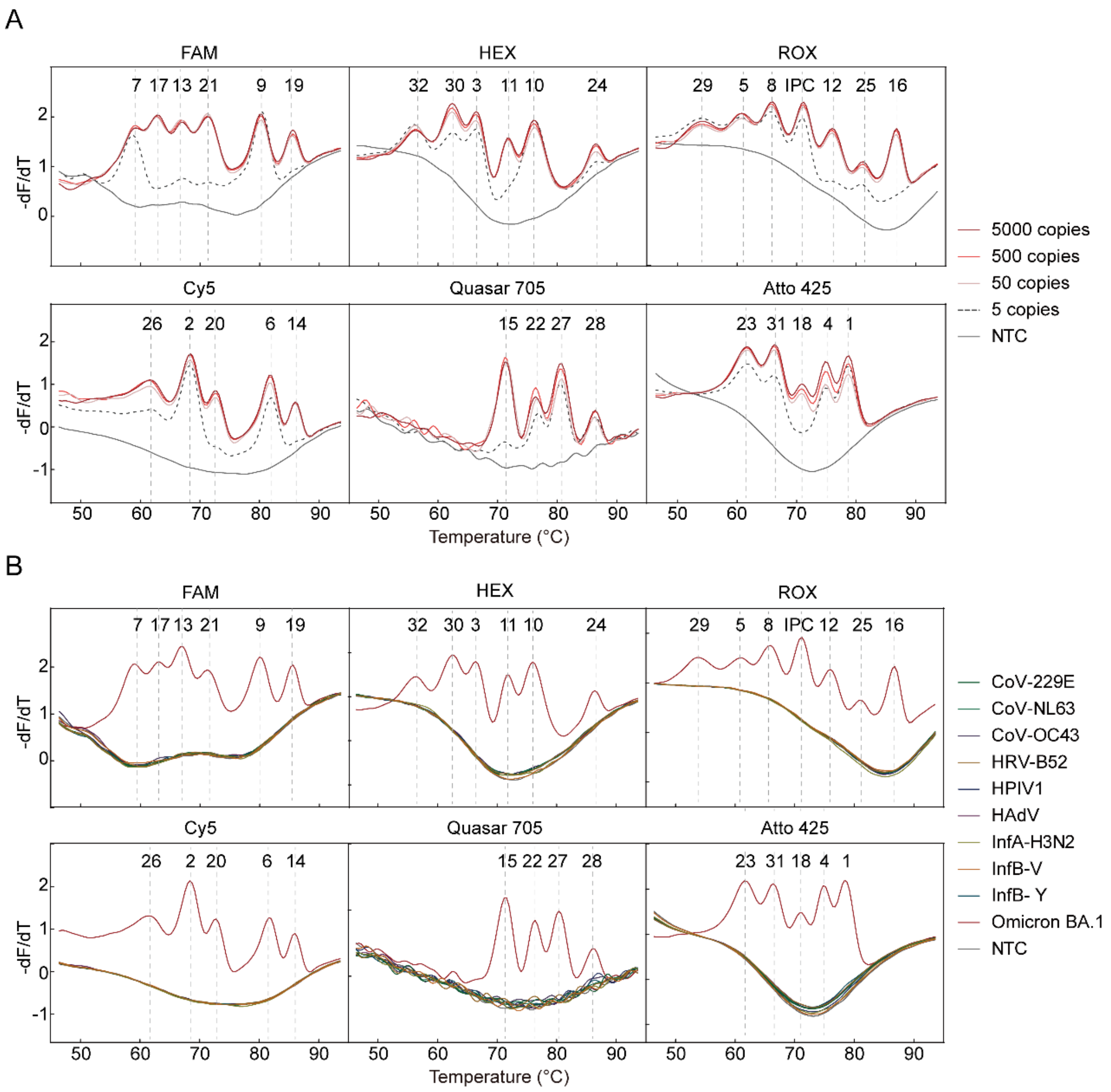

Fig. S1. Analytical performance of the 33-target MeltArray assay. (A) The limit of detection (LOD) of the 33-target MeltArray assay. 10-fold serial dilutions of the Omicron BA.1 sample ranging from 5 to 5000 copies/reaction were detected. Gray lines indicate no-template control (NTC). (B) Cross-specificity of the 33-target MeltArray assay. Omicron BA.1 and nine other respiratory viruses were used as templates, no cross-reactivity was observed.
