# Supplementary material for: Accessible and Adaptable Multiplexed Real-Time PCR Approaches to Identify SARS-CoV-2 Variants of Concern
Source: Microbiol Spectr. 2022 Sep 15;10(5):e03222-22. doi: 10.1128/spectrum.03222-22 (PMC9603638; doi:10.1128/spectrum.03222-22)
Supplement: Reviewer comments [file reviewer-comments.pdf]

AMERICAN SOCIETY FOR MICROBIOLOGY | Microbiology Spectrum

Accessible and Adaptable Multiplexed Real-Time PCR Approaches to Identify SARS-CoV-2 Variants of Concern

Ting Yan, Ye Xu, Rongrong Zheng, Xiaohong Zeng, Zehui Chen, Su Lin, Zihan Xia, Yiqun Liao, Yongyou Zhang, and Qingge Li

Corresponding Author(s): Qingge Li, Xiamen University

Review Timeline:

Submission Date:	August 26, 2022
Accepted:	September 1, 2022

Editor: Tulip Jhaveri

Reviewer(s): The reviewers have opted to remain anonymous.

Transaction Report:

(Note: With the exception of the correction of typographical or spelling errors that could be a source of ambiguity, letters and reports are not edited. The original formatting of letters and referee reports may not be reflected in this compilation.)

DOI: https://doi.org/10.1128/spectrum.03222-22

September 1, 2022

Prof. Qingge Li
Xiamen University
School of Life Sciences
Huangchaoyang Buliding E202
Xiang'an Campus Xiamen University
Xiamen, Fujian 361102
China

Re: Spectrum03222-22 (Accessible and Adaptable Multiplexed Real-Time PCR Approaches to Identify SARS-CoV-2 Variants of Concern)

Dear Prof. Qingge Li:

Your manuscript has been accepted, and I am forwarding it to the ASM Journals Department for publication. You will be notified when your proofs are ready to be viewed.

The ASM Journals program strives for constant improvement in our submission and publication process. Please tell us how we can improve your experience by taking this quick Author Survey.

As an open-access publication, Spectrum receives no financial support from paid subscriptions and depends on authors' prompt payment of publication fees as soon as their articles are accepted. You will be contacted separately about payment when the proofs are issued; please follow the instructions in that e-mail. Arrangements for payment must be made before your article is published. For a complete list of **Publication Fees**, including supplemental material costs, please visit ourwebsite.

ASM policy requires that data be available to the public upon online posting of the article, so please verify all links to sequence records, if present, and make sure that each number retrieves the full record of the data. If a new accession number is not linked or a link is broken, provide production staff with the correct URL for the record. If the accession numbers for new data are not publicly accessible before the expected online posting of the article, publication of your article may be delayed; please contact the ASM production staff immediately with the expected release date.

Corresponding authors may join or renew ASM membership to obtain discounts on publication fees. Need to upgrade your membership level? Please contact Customer Service at Service@asmusa.org.

Thank you for submitting your paper to Spectrum.

Sincerely,

Tulip Jhaveri
Editor, Microbiology Spectrum

Journals Department
American Society for Microbiology
1752 N St., NW
Washington, DC 20036
E-mail: spectrum@asmusa.org

Supplemental Material: Accept
